# 

**Published:** 1823-06-01

**Authors:** 


### Medico-Chirurgical Review,

AND

### JOURNAL

OF

### MEDICAL SCIENCE.

(Quarterly.)

EXHIBITING A COMPREHENSIVE ANALYTICAL RECORD OF PROGRESSIVE MEDICINE AND SURGERY;

*EQUALLY ADAPTED TO ALL RANKS OF THE PROFESSION.*

CONDUCTED BY
ASSOCIATED PHYSICIANS AND SURGEONS;

AND SUPERINTENDED BY
JAMES JOHNSON, M. D.
MEMBER OF THE ROYAL COLLEGE OF PHYSICIANS OF LONDON.

(Analytical Series.)

---

VOLUME IV. for 1823-4.

---

Nec tibi quid liceat sed quid fecisse decebit
Occurrat mentemque domat respectus honesti. CLAUD.

---

London:
Printed by G. HAYDEN, Little College Street, Westminster,
FOR THE EDITOR,
No. 58, Spring Gardens,
[Where Books for Review and Critical Communications, &c. *(post paid)* are, to be directed.]
Published by BURGESS & HILL, Windmil Street, Haymarket; BALDWIN CRADDOCK, & JOY, Pater-Noster Row; PARBURY, & Co. Leadenhall Street; HIGHLEY, and T. & G. UNDERWOOD, Fleet Street; CALLOW and WILSON, Princes Street, Soho; COX and SON, Borough; JACKSON, Borough; ANDERSON, West Smithfield; JOHN COX, Berners Street, Oxford Street; ADAM BLACK, Edinburgh; RICHARD GRIFFIN & Co. Glasgow; HODGES and M'ARTHUR, Dublin; Messrs. TREUTTEL and WURTZ, Paris and Strasburgh.

1824.

W1
ME75Q

### XIII.

### BIBLIOGRAPHICAL RECORD;

OR

*Works received for Review since last Quarter.*

1. A Discourse on Medical Education, and on the Medical Profession. By JOHN G. COFFIN, M.D. Fellow of the Massachusetts Medical Society. Octavo, pp. 46. Boston, 1822.

☞ *We agree perfectly with Dr. Coffin, in the enlightened views which he has taken of the two subjects abovementioned. Would that they were practically acted on, both East and West of the Atlantic.*

---

2. A brief Description of the salutary Effects of Chlorine, as administered by Mr. Charles Troward, at the Fumigating Establishment, 25, Red Lion Square.

---

3. Reply to a Pamphlet, entitled "Notes on Dr. Mackintosh's Treatise on Puerperal Fever, by Mr. Moir, Surgeon," and to the Letters in the Appendix, by Dr. James Hamilton, Jun. By JOHN MACKINTOSH, M.D. Octavo, pp. 82. Edinburgh and London, 1823.

☞ *Dr. Mackintosh is milder in this Reply than we could have well expected, after such a scurrillous and libellous assault. He more than insinuates, (what we have great reason to think is true,) that Dr. Hamilton is the real author of Mr. Moir's pamphlet.*

---

4. Practical Observations on Fever, Dysentery, and Liver Complaints, as they occur amongst the European Troops in India; illustrated by numerous Tables and Cases. To which is annexed, an Essay on Syphilis. By GEORGE BALLINGALL, M.D. F.R.S. E. Surgeon Extraordinary to the King for Scotland, Regius Professor of Military Surgery in the University of Edinburgh, &c. &c. *Second Edition.* Octavo, pp. 305. Edinburgh and London, 1823.

☞ *We are happy to announce a new and improved edition of Dr. Ballingall's very valuable work. It has been eagerly inquired for; and forms an essentially necessary part of the tropical practitioner's library. We have not ceased, and shall not cease, to recommend it in the strongest terms to our brethren preparing for our Eastern colonies. Our readers are aware that we have given an extensive review of the Work in a former number of this Journal.*

---

5. Remarks on the Yellow Fever of the South and East Coasts of Spain; comprehending Observations made on the Spot, by actual Survey of Localities, and rigorous Examination of Fact at original Sources of Information. By THOMAS O'HALLORAN, M. D. Member of the Medical Academy of Madrid and Barcelona. Octavo, pp. 208, with a large Chart of Barcelona, price 10s. 6d. boards. Callow and Wilson, 1823.

---

6. Remarks and Practical Results of Observation on Scirrhus and Cancer. By ANTONIO SCARPA; translated from the Italian, with an Appendix, containing an Account of a Sanguineous Tumour of the Breast, and some Annotations on the Text. By JAMES BRIGGS, Surgeon to the Public Dispensary, and Assistant Surgeon to the Lock Hospital. Octavo, sewed, pp. 62, with a plate. London, 1823.

---

7. Tentamen Medicum inaugurale de Sephilitide. Auctore S. HILL, M.D. Edinburgh, 1822.

---

8. The Utility and Importance of Fumigating Baths illustrated; or, a Series of Facts and Remarks, shewing the Origin, Progress, and Final Establishment (by order of the French Government) of the Practice of Fumigation for the Cure of various Diseases, &c. &c. By JONATHAN GREEN, Member of the Royal College of Surgeons London, and late Surgeon in His Majesty's Navy. 8vo, sewed, pp. 115. London, 1823.

---

9. System of Anatomical Plates, with descriptive Letter-press. By JOHN LIZARS, F.R.S. Fellow of the Royal College of Surgeons; and Lecturer on Anatomy and Physiology, Edinburgh. Part I. The Bones. Eight plates, folio; and 119 pages of letter-press, price 10s. 6d.

☞ *The great aim of the author being the most scrupulous correctness, he has either drawn the different objects himself, or superintended the drawings made by his brother; so that by the pencil of the one, and the careful superintendence of the other, he trusts the plates will be found worthy of examination. To obviate expense, the letter-press has been printed in octavo size, which, in addition to the advantage of cheapness, will also be found more convenient for reference. The anatomy is blended with physiology and pathology, so as to prove interesting and instructive. We have no hesitation in pronouncing the undertaking to be a work of merit; and, from its cheapness, well deserving the attention of the anatomical student in particular.*

---

10. Archio fur Medizinische Erfahrung im Gebiete der Praktischen Medizin, Chirurgie, Geburtshulfe und Staatsarzneikunde. Herausgegeben von Dr. HORN in Berlin, Dr. NASSE in Bonn, Dr. HENKE in Erlangen, und Dr. WAGNER in Berlin. Julius, August 1822. *In exchange for Medico-Chirurgical Review.*

---

11 Thoughts on the present character and constitution of the Medical Profession. By T. C. SPEER, M.D. M.R.I.A. late Physician to the Dublin General Dispensary, &c. Octavo, pp. 132. Cambridge and London, 1823.

---

12. Shampooing; or, Benefits resulting from the Use of the Indian Medicated Vapour Bath, as introduced into this Country by S. D. Mahomed, (a native of India;) containing a brief but comprehensive View of the Effects produced by the Use of the Warm Bath, in comparison with the Steam or Vapour Bath, &c. &c. By LAKE DEAN MAHOMED. Octavo, pp. 127. Brighton, 1823.

---

13. Elements of the Theory and Practice of Physic, designed for the Use of Students. By GEORGE GREGORY, M.D. Licentiate of the Royal College of Physicians of London, and Senior Physician to the St. George's and St. James's Dispensary. In two volumes. Volume the Second, pp. 450. London, April 1823.

14. The Elements of Pharmacy, and of the Chemical History of the Materia Medica, &c. &c. intended as a Companion to the Author's Treatise on Pharmacology. By SAMUEL FREDERICK GRAY, Lecturer on Materia Medica, Botany, and Pharmaceutic Chemistry. Octavo, pp. 296. London, 1823.

---

15. An Exposition of the Principles of Pathology, and of the Treatment of Diseases. By DANIEL PRING, M.D. Member of the Royal College of Surgeons of London. Octavo, pp. 512. Underwoods, London, 1823.

---

16. A short Treatise on Operative Surgery, describing the principal Operations as they are practised in England and France; designed for the Use of Students, in operating on the Dead Body. By CHARLES AVERILL, Surgeon, Cheltenham. Octavo, pp. 172. Jackson, London. May 1823. Price 6s. boards.

☞ *The design of this little Work is good, and the execution very fair. No concise work exists, the sole object of which is to enable the student to practise surgical operations upon the dead subject, preparatory to performing them on the living. This desideratum is attempted in the present little work. The modes of operating, at present in use in England, and on the Continent, are concisely and perspicuously stated; and several of the operations are prefaced by historical remarks, which strikingly exemplify the improved state of surgery. The whole is arranged in a manner consistent with the author's wish of presenting the greatest number practicable upon one dead body. We think this little work will be a useful companion in the dissecting room, and we can recommend it accordingly. It is dedicated,* by permission, *to Sir Astley Cooper. We shall notice some passages in our next number.*

---

17. A Practical Treatise on the Symptoms, Causes, Discrimination, and Treatment of some of the most important Complaints that affect the Secretion and Excretion of the Urine, &c. &c. exhibiting a comprehensive View of the various Diseases of the Kidneys, Bladder, Prostate Gland, and Urethra. Illustrated by numerous Cases and Engravings. By JOHN HOWSHIP, Member of the Royal College of Surgeons in London, &c. Octavo, pp. 438, four plates. London, April 1823.

☞ *This very valuable Work in our next.*

---

18. A Series of Elementary Lectures on the Veterinary Art; wherein the Anatomy, Physiology, and Pathology of the Horse are essayed on the general Principles of Medical Science. By Veterinary Surgeon PERCIVAL, of the Royal Regiment of Artillery. Octavo, pp. 377.

☞ *The diseases of horses are almost as interesting to the medical practitioner as those of the human subject—for the horse is his daily and faithful companion. We recommend this Work to our brethren, and we shall give some account of the performance in our next number.*

---

19. A Letter to the Right Honorable the Lord Chancellor, on the Nature and Interpretation of Unsoundness of Mind, and Imbecility of Intellect. By JOHN HASLAM, M.D. late of Pembroke Hall, Cambridge. Octavo, sewed, pp. 32. May 1823.

### TO CORRESPONDENTS, &c.

The letter dated March 28th, and signed "A friend and ———," has been duly received, and for which thanks are hereby returned. There is but one point requiring an answer in this place. The editor is *not* the author of the article commented on.

Mr. Peter Donalson, Chirurgeon of New York, has put us to the expense of 1*l*. 8*s*. 6*d*. postage, for a pamphlet not worth one farthing. We must, in future, refuse all packets from America, and other foreign parts, that are presented to us through the medium of the post office.

In compliance with the wishes of several subscribers, we are preparing a review of Pearson's life of Hey, which will probably appear in our next.

In answer to several correspondents, who regret that so important a department as the Periscope should ever be omitted in the Journal, we have to state that arrangements are made to ensure the regular appearance of the Periscope in future. It was the wish to bring up the arrears of the review department at the close of the volume, which occasioned the omission in our last number.

The extraordinary epistle of "Economicus" has come to hand. We perceive that, with him, economy begins at home—for he left unpaid the postage of his letter. This, however, is not the most provoking part. He accuses us of driving a hard bargain with the public, and attaching a high price to our work. If Economicus be acquainted with the rudiments of arithmetic, he will find, on calculation, that the established limits of the Journal (14 sheets, or 224 pages) are somewhat below (not above) the ratio of price exacted by our cotemporaries. And excepting in one or two numbers, the Journal has regularly exceeded the specific limits by from eight to twenty-four or thirty pages of letter-press. If this be driving a hard bargain, it is wondrous strange. We beg to inform Economicus, therefore, that we will not commence the depreciation of our own labours till the public withholds its patronage —of which there are no very strong symptoms as yet manifested.

The review of ———, by "Judex," came to hand, but cannot be made use of. There is evidently private or personal feeling mixed up with the criticisms—a feeling which we never will admit into our Journal, when we can detect it. Tart criticism is doubtless agreeable to the jaded appetite, but frigid analysis is more wholesome for the hungry healthy stomach.

To Philosophus we return thanks for his friendly advice, though we do not deem it prudent to follow it. Our's is an extremely limited monarchy, the course of our duty being very clearly defined—that of presenting as comprehensive yet concentrated a portrait of the current literature and science of the day as our pages will admit. To trend towards any particular department of science—and especially towards that pointed out by Philosophus, would be to disturb the salutary and natural balance of the whole. We do not pretend to divine, and we cannot expect to suit the tastes of all readers—but Philosophus may take it for granted that we can give a shrewd guess at that which pleases the majority—the minority must please themselves.

Scoto-Severus must be clean daft. Does he suppose we can compel men to write good books, or prevent their publishing bad ones? To praise the one or censure the other, without offering materials whereon the judgment of the reader himself might be founded, would be at direct variance with the fundamental principle of this Journal. And hence our individual numbers must vary in interest with the chance of the day. There are few publications, however mediocre, from which we cannot extract some useful matter; and, at all events, it would not be for the interest of the public to pass over entirely unnoticed the inferior productions of the press. By exhibiting specimens of all, the reader is put in possession of the means of judging what he shall place in his library, and what not.

The able review of the different modern theories of fever, drawn up in the intellectual city, has been perused, but we dare not venture on its publication, at least at present. The public mind is in a state of *apyrexia*, and we should be loth to exhibit such powerful *antiphlogistic articles* till some symptoms of *excitement* reappear.

The communication from Dr. Bull, of Cork, has been properly disposed of.

---

### *Additional Subscribers since last Quarter.*

Averill, Charles, Esq. Fellow of the Royal College of Surgeons, Cheltenham
Anderson, Chas. Esq. to Bengal
Beddome, Mr. Surgeon, Romsey
Baker, James, Esq. Colchester
Bull, Dr. Christopher, Aldworth, Surg. to the South Infirmary, and Teacher of Anatomy, Cork
Baker, Mr. (residence not stated)
Creaser, Dr. Cheltenham
Cathral, Mr. George
Dods, Dr. Andrew, Bath
Dewar, Mr. Andrew, Surgeon, Dumfermline, N. B.
Dennison, Charles, M.D.
Elkington, F. Esq. Surg. M.C.S. Paul Square, Birmingham
Ellis, George, Esq.
Finlayson, Robert, M. D. Member of the Royal College of Surgeons of London, and Surgeon in the Royal Navy
Fowler, Dr. Robert, to West Indies
Griffith, Mr. Surgeon, Holyhead
Glasgow Medical Library
Gilbert, Dr. Paris
Holt, Geo. Palmer, M.D. Physician to the Essex and Colchester Hospital
Harland, C. Esq. Ashbourne
Hannah, Mr. James, Surg. H. M. Cutter, Swan
Heaviside, William, Esq.
Johnston, Geo. M. D. Berwick-upon-Tweed
Johnstone, Dr. G. W. Cockermouth
Joseph, Charles, M.D.
Kennedy, Mr. Thos. (residence not stated)
Laves, Mr. Charles-street, Westminster
Love, Mr. (Student)
Malta Military Medical Library
Med.-Chirurgical Society, Hexham, Northumberland
M'Kenzie, Mr. John, H. S. I. Inverness
Murley, Mr. S. H. Surg. Cheltenham
Menzies, George, Esq.
Norwood, Mr. Peter
Perks, Mr. Watson, Surgeon, Hitchin
Quintin, Mr. George, to India
Robertson, William, M. D.
Smith, Mr. William Bestoe, Surgeon, Ludbury
Sleight, Mr. R. P. Surg. Hull
Simpson, Dr. Bradford, Yorkshire
Simmonds, Mr. B. H. Surgeon, Sherborne
Tityres, Mr. Member of the Royal College of Surgeons, Stockton-on-Tees
Valentine, Mr. Surg. Somerton, Somersetshire
Winter, Mr. Surgeon, Romsey
Wagner, Dr. Berlin
Williams, P. Esq. Rugeley
Woodforde, Dr. Castle Cray, Somerset
Yonge, Mr. W. Member of the Royal College of Surgeons, St. Ives, Cornwall.

